# Boosting Confidence: Enhancing Spinal Cord Stimulator Needle and Lead Placement Through Simulation Training

**DOI:** 10.7759/cureus.55550

**Published:** 2024-03-05

**Authors:** Shan Ali, Mark Friedrich Hurdle, Salim M Ghazi, Sahil Gupta

**Affiliations:** 1 Neurology, Mayo Clinic, Jacksonville, USA; 2 Pain Medicine, Mayo Clinic, Jacksonville, USA

**Keywords:** neuromodulation, procedural confidence, medical education, resident training, fellowship training, spinal cord stimulation

## Abstract

Background

This pilot study aims to examine the effectiveness of a spinal cord stimulator (SCS) simulator training system in improving the confidence of pain fellows in SCS placement.

Methodology

Five Ukrainian physicians (neurologists, neurosurgeons, and an anesthesiologist) completed a 10-item survey regarding their confidence in various aspects of SCS placement and their opinions on how effective SCS models were for educational purposes. After placing SCS leads using the SCS simulator, the physicians took the same survey again. The Mann-Whitney U test was used to determine if there was a significant difference in total scores pre and post-simulator training. The software PAST (PAleontological STatistics) was used for statistical analysis.

Results

Overall, five participants had a 38% statistically significant increase in survey scores before and after the intervention (mean: 4.2 vs. 6.2, p = 0.0055). With regards to each item of the survey, participants had a significantly increased confidence in driving leads (2.6 vs. 5.2, p = 0.008) and in overall technical skills for the SCS procedure after the training (2.8 vs. 5.2, p = 0.0188). Although the other eight survey items were not statistically significant (p > 0.05), participants had a 28% increase in confidence when inserting epidural needles, a 20% increase in interpreting simulated X-rays, a 32% increase in navigating challenging anatomical variations, a 12% increase in identifying key anatomical landmarks, a 20% increase in ensuring the correct placement of the lead, or a 53% increase in preparedness for performing an SCS procedure in a real clinical setting. The participant’s perspective on how valuable the stimulator training was for enhancing procedural skills increased by 38% and how well the simulator replicated real-life SCS procedure increased by 52%, although both were statistically insignificant (p > 0.05).

Conclusions

This pilot study shows that the utilization of simulated neuromodulation training is a viable means of augmenting neuromodulation education by increasing physician’s confidence in aspects of the SCS placement procedure. The extent to which simulator training improves procedural skills in a real-life SCS placement needs to be investigated further.

## Introduction

Traditional training methods for fluoroscopically guided procedures involve the use of cadavers with live fluoroscopy or participation in clinical encounters. Human cadavers have limitations such as morbidity, cost, and limited durability. In addition to the above, the tactile feel in cadavers can be inaccurate depending on a multitude of factors. The utilization of live fluoroscopy in training poses inherent health risks associated with radiation exposure, even within a controlled educational environment [1]. Ionizing radiation is harmful due to its ability to damage DNA and cause tissue damage and cancer, with cumulative effects over time. Medical professionals follow the “as low as reasonably achievable” principle to minimize radiation exposure for both patients and healthcare workers. To monitor exposure to ionizing radiation healthcare providers wear dosimeters to estimate the operator’s effective dose. The total fluoroscopy time for a percutaneous spinal cord stimulator (SCS) trialing procedure is about 133 seconds [2] and fellows performing interventional radiology procedures typically receive about 31% higher radiation doses than a fully trained physician [3]. Moreover, pain fellowship programs often do not have enough cases for pain fellows to feel comfortable in placing SCS. As of 2017, 46% of pain fellows in the United States felt that there was an unmet training need regarding SCS, with the majority attributing it to a lack of SCS case volume and needing to supplement their learning by participating in educational programs from SCS manufacturers [4]. The COVID-19 pandemic also had a detrimental effect on SCS training of Pain Medicine fellows. The pandemic resulted in postponed or canceled SCS procedures, reducing pain medicine fellows’ hands-on training during the initial shutdown [5]. This interruption intensified challenges in pain fellows’ transition to attending physicians with even fewer SCS trials and implant experience. With health systems working to return to normalcy amid ongoing uncertainty from variant strains, ensuring technical proficiency through guaranteed procedural repetitions remains a challenge.

Three-dimensional (3D) SCS simulator training systems exist as a potential solution to mitigate the lack of exposure and training to SCS implantation [6]. These training systems have the advantage of providing fellows with unlimited training repetitions, no radiation exposure, and immediate feedback.

However, there is a paucity of research regarding the utility of these 3D training systems in helping trainees’ confidence regarding SCS implantation and increasing their SCS implantation skills. To date, only one small single-center study has evaluated the utility of a 3D SCS simulator training system to help increase the confidence of training physicians [6]. This 14-person study found that virtual SCS simulation helped trainees improve their confidence with interlaminar epidural access and SCS placement overall, as well as improve their procedural skills [6].

In this pilot study, we continue to determine the effectiveness of a 3D SCS simulator training system in improving pain fellows’ confidence in SCS placement by using a survey to measure the pre- and post-confidence levels. We also examine the perceived usefulness of stimulator training pre- and post-intervention. The information presented in this study will help support the use of 3D SCS simulator training systems in medical education. Incorporating a 3D simulation training tool for neuromodulation may improve participants’ confidence and proficiency in epidural access and SCS placement.

## Materials and methods

Study population

Five voluntary participants were recruited from Kyiv Medical University Hospital in Ukraine who had graduated from residency and had never placed an SCS before, observed a real-life neuromodulation case, and had no prior experience with SCS simulators. The study was conducted at Mayo Clinic in Jacksonville Florida, an academic medical center, during October 2023. Participants from Kyiv Medical University Hospital in Ukraine were selected based on the alignment of their specific needs with the research objectives.

Simulator

**Figure 1:**
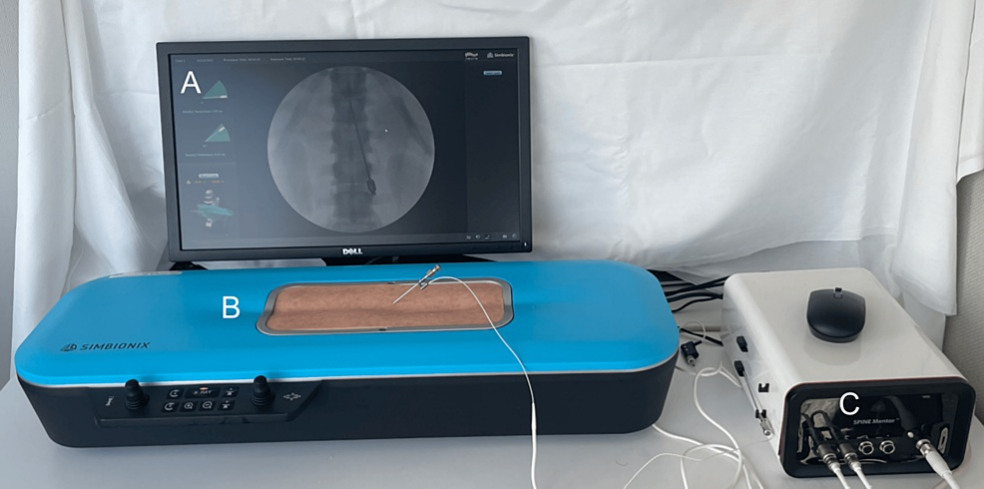
Spinal cord stimulator implantation simulator (the Spine Mentor). A: A visual display of simulated fluoroscopic images emulating a virtual C-arm. B: A puncture pad with an anatomically correct spine underneath. C: Computer box.

We employed the SPINE Mentor™ (Simbionix Ltd, Surgical Science Sweden AB) to simulate the visual and tactile sensation of a live SCS implantation procedure, which can be seen in Figure 1. The simulator employed a virtual C-arm, a syringe with a loss of resistance sensor, a puncture pad, and dynamic haptics to simulate anatomical obstacles. This tool integrated a 3D spine model and virtually simulated fluoroscopic images. The margin of error with this instrument was not published by the manufacturer.

Study protocol

This study is a prospective cohort study. Before engaging in the simulation, participants were administered a 10-item pre-intervention survey aimed at gauging their confidence levels in the placement of SCS and view of the 3D simulator as a method of education on SCS implantation. Each item’s assessment was based on a scale ranging from 1 to 10. Following the survey, each participant underwent a brief introduction to the hardware and its components, followed by specific instructions to place an SCS lead using an interlaminar approach between T12 and L1, with the electrodes advancing to the level of T8 to T9. Afterward, the participant took a post-intervention survey that was identical to the pre-intervention survey (see Appendices).

This study involved the use of a simulation model to educate physicians on gaining epidural access and driving SCS leads, focusing exclusively on the development of technical skills within an educational setting. Given the nature of the study, where direct patient interaction or intervention was not involved, and the research activities were confined to the use of non-human models for procedural training, institutional review board (IRB) approval was not necessary. This determination is consistent with federal regulations exempting certain categories of research from IRB review, particularly those involving educational practices designed to improve professional skills without involving patients or sensitive data. Furthermore, the study ensured that there was no risk to human subjects, aligning with the criteria for exemption as it did not involve collecting or analyzing identifiable private information from or about patients.

Statistical methods

Descriptive statistics for continuous variables covered mean and range. The Shapiro-Wilk test was used to test for normality. The Mann-Whitney U test was used to find differences between pre- and post-intervention survey scores. The non-parametric Mann-Whitney U test was used rather than the t-test because of the small sample size and lack of normal distribution. A p-value <0.05 was deemed significant. The software PAST which stands for PAleontological STatistics (Hammer and Harper, Øyvind Hammer, Natural History Museum, University of Oslo, Oslo, Norway) was used for statistical analysis.

## Results

**Figure 2:**
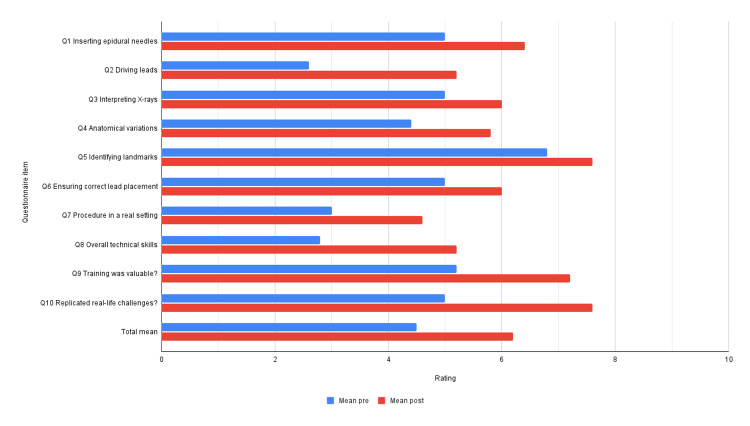
Pre- and post-simulator mean confidence levels regarding spinal cord stimulator placement.

Five participants were recruited, including two neurologists, one anesthesiologist, and two neurosurgeons. Each participant filled out and returned the survey before and after the intervention. Figure 2 illustrates the mean pre- and post-intervention score.

Increase in survey scores

Participants exhibited a substantial 38% statistically significant increase in survey scores following the intervention, demonstrating a notable improvement in their overall performance (mean: 4.2 vs. 6.2, p = 0.0055).

Enhanced confidence in specific tasks

Participants demonstrated significantly heightened confidence levels in key tasks post-intervention, particularly in driving leads (2.6 vs. 5.2, p = 0.008) and in mastering the technical skills required for the SCS procedure (2.8 vs. 5.2, p = 0.0188).

Improvements in noteworthy skills

Although not all individual survey items reached statistical significance (p > 0.05), participants displayed increased confidence across multiple domains. This included a 28% increase in confidence regarding epidural needle insertion, a 20% increase in interpreting simulated X-rays, a 32% increase in navigating challenging anatomical variations, a 12% increase in identifying key anatomical landmarks, a 20% increase in ensuring correct lead placement, and a 53% increase in preparedness for performing SCS procedures in real clinical settings.

Perceived value of stimulator training

Participants reported a notable 38% increase in their perception of the value of stimulator training for enhancing procedural skills, underscoring the perceived efficacy of the intervention.

Simulator fidelity

Participants’ assessment of the simulator’s replication of real-life SCS procedures increased by 52%. Though statistically insignificant (p > 0.05), the perceived fidelity of the simulation experience was increased.

## Discussion

We found that using a 3D SCS training model significantly increased participants’ confidence in lead placement during SCS trials and implants and in overall technical skills for the SCS procedure after the training. Therefore, virtually simulated neuromodulation training models may improve the confidence that 46% of pain fellows feel due to low case volume exposure, decrease the reliance on educational programs from SCS manufacturers, and increase the willingness of pain fellow graduates to offer SCS to their patients when practicing independently [4]. As 20.5% of adults in America experience chronic pain, it is critical that SCS implantation is offered when appropriate because it may reduce pain, reduce opiate use, promote the return to work from disability, and help avoid major back surgeries [7-9]. This study is novel as it is the first follow-up study to evaluate the 3D SCS training simulator model with a diverse background of physician participants outside of the United States [6].

Our study showed that participants reported a mean 86% increase in their procedural confidence with SCS post-intervention. White et al. showed similar findings in their 14-physician study using the same 3D SCS training model [6]. They found that the mean confidence levels improved by 71.4% for interlaminar epidural access and 306% for SCS placement.

The strength of this study includes the implementation of pre-intervention and post-intervention surveys, which specifically targeted various aspects of SCS stimulator placement and the perceived value of stimulator training which had not been thoroughly analyzed previously [6]. Furthermore, the study population consisted of participants with no prior SCS placement experience. This ensured that previous exposure to SCS placement techniques did not influence the results, thereby reducing potential confounding variables and enhancing the validity of the findings. The study’s focus on individuals without prior experience also reflects the potential applicability of simulator-based training programs to novice practitioners who are in the early stages of their medical careers.

The cost of the simulation device used in this paper is $50,000 which may deter pain medicine programs from using it in their fellow’s education [6]. However, inexpensive 3D spine phantoms can be made by physicians for pain medicine procedure training for as little as $13 using 3D printers [10]. Design files, recommended materials, and a step-by-step manual for making 3D spine phantoms are available for download online [10,11]. Koh et al. created a 3D-printed lumbar spine model from a patient’s computed tomography and magnetic resonance imaging data for teaching selective transforaminal epidural block, medial branch block, and lumbar sympathetic ganglion block procedures [12]. They found that participants who received training using the 3D phantom had a decrease in the number of C-arm images taken and an increase in the global rating score than participants who did not [12]. The global rating score they used was based on a seven-item (five-point scale tool) measuring participants’ respect for tissue, time and motion, instrument handling, fluoroscopic handling, use of assistants, the flow of procedure, and forward planning and knowledge of the procedure.

This study has several limitations. There was not enough statistical significance for several of the survey items pre- and post-intervention, likely due to the limited number of participants (n = 5). Moreover, this study qualitatively measured the confidence of the operator via a survey and did not objectively measure procedural skills. With regards to the 3D simulator itself, real-life encounters may have additional factors that may decrease the generalizability of the device. For example, increased adipose tissue and degenerative spine changes may make access to the epidural space more difficult. Additionally, the quality of the C-arm used and the level of visual and tactile feedback available can be considerably different with an SCS simulator when compared with a real-life patient. This study recorded and analyzed only the pre- and post-intervention surveys. However, including the participants’ subjective perspectives of the simulator in a narrative may augment the study’s findings and bring up issues that were previously missed.

Future directions of this study include longitudinal assessments to gauge the durability of confidence and skill enhancement post-simulator training to determine if improvements translate into sustained benefits and improved clinical outcomes. Validation of the SCS simulator against clinical benchmarks and outcomes would establish the credibility and effectiveness of the simulation technology. Conducting cost-effectiveness analyses would offer insights into the economic impact of simulator-based training compared to traditional methods, informing resource allocation and investment decisions. Finally, integrating simulation training into residency and fellowship programs would ensure standardized and comprehensive education in SCS placement, preparing future healthcare professionals for clinical practice and fostering a culture of continuous learning and skill development.

Conventional training modalities for physicians, including cadavers with live fluoroscopy or clinical encounters, have drawbacks such as cost and limited durability. Moreover, live fluoroscopy carries health risks from radiation exposure, necessitating alternative SCS placement training methods. The COVID-19 pandemic exacerbated challenges in pain fellows’ training by causing postponed or canceled SCS procedures, reducing hands-on experience, and complicating their transition to attending physicians, impacting patient care and SCS adoption. Our study evaluates a 3D SCS simulator’s effectiveness in enhancing pain fellows’ confidence and technical skills in SCS placement. The significant increase in participants’ confidence levels in key tasks post-intervention underscores the potential of simulator-based training to address limited case volumes and interruptions such as the pandemic. By fortifying trainees’ confidence and technical skills in SCS placement, our study emphasizes the importance of integrating 3D simulation tools into pain medicine fellowship programs.

## Conclusions

Our pilot study demonstrates a notable enhancement in trainee confidence following the implementation of a 3D SCS simulator training system. The statistically significant increase of 38% in overall survey scores, coupled with specific improvements in key areas such as driving leads and mastering technical skills for the SCS procedure, underscores the efficacy of this training approach. The substantial percentage increases in confidence across various procedural aspects highlight the potential of the simulator to bolster trainees’ preparedness for real clinical settings. Moreover, participants’ perspectives on the value and fidelity of the simulator training showed promising upward trends. Our findings support the integration of 3D simulation tools into pain medicine fellowship programs, ultimately equipping trainees with the confidence necessary for successful clinical practice in neuromodulation. By bolstering physicians’ confidence in SCS placement, 3D SCS simulator training systems have the potential to mitigate the impact of limited SCS case volumes, diminish reliance on educational initiatives provided by SCS manufacturers, and, consequently, broaden the accessibility of SCS treatment options for patients.

## Appendices

Answer the following questions on a scale of 0 to 10 with 0 meaning “not at all” and 10 meaning “definitely yes.”

**Table 1:** Pre- and post-simulator training questionnaire. SCS = spinal cord simulation

Number	Question	0 to 10
1	How confident are you in inserting epidural needles for SCS?	
2	How confident are you in driving leads for SCS?	
3	How confident are you in interpreting simulated X-rays during the procedure?	
4	How well do you feel you can navigate challenging anatomical variations like scoliosis, obstructions in the epidural space, etc.?	
5	How confident are you in identifying key anatomical landmarks during the procedure?	
6	How confident are you in ensuring the correct placement of the lead?	
7	How prepared do feel to perform an SCS procedure in a real clinical setting?	
8	How confident are you in your overall technical skills for the SCS procedure after the training?	
9	How valuable do find the stimulator training for enhancing your procedural skills?	
10	How well do you feel the stimulator replicates real-life SCS procedural challenges?	
